# Sexual selection modulates genetic conflicts and patterns of genomic imprinting

**DOI:** 10.1111/evo.13153

**Published:** 2017-01-16

**Authors:** Gonçalo S. Faria, Susana A. M. Varela, Andy Gardner

**Affiliations:** ^1^ School of BiologyUniversity of St AndrewsDyers Brae, St AndrewsKY16 9THUnited Kingdom; ^2^ cE3c—Centre for Ecology, Evolution and Environmental Changes, Faculdade de CiênciasUniversidade de LisboaCampo Grande1749‐016LisboaPortugal

**Keywords:** Arms race, dispersal, inclusive fitness, intragenomic conflict, kin selection, sexual conflict

## Abstract

Recent years have seen a surge of interest in linking the theories of kin selection and sexual selection. In particular, there is a growing appreciation that kin selection, arising through demographic factors such as sex‐biased dispersal, may modulate sexual conflicts, including in the context of male–female arms races characterized by coevolutionary cycles. However, evolutionary conflicts of interest need not only occur between individuals, but may also occur within individuals, and sex‐specific demography is known to foment such intragenomic conflict in relation to social behavior. Whether and how this logic holds in the context of sexual conflict—and, in particular, in relation to coevolutionary cycles—remains obscure. We develop a kin‐selection model to investigate the interests of different genes involved in sexual and intragenomic conflict, and we show that consideration of these conflicting interests yields novel predictions concerning parent‐of‐origin specific patterns of gene expression and the detrimental effects of different classes of mutation and epimutation at loci underpinning sexually selected phenotypes.

Recent years have seen a surge of interest in the interplay of kin selection and sexual selection (Boomsma 2007; Rankin 2011; Wild et al. 2011; Pizzari and Gardner 2012; Carazo et al. 2014; Chippindale et al. 2015; Faria et al. 2015; Hollis et al. 2015; Martin and Long 2015; Pizzari et al. 2015). A key theme is the potential for relatedness within mating groups to reduce sexual conflicts. For example, Rankin's (2011) theoretical analysis suggests that kin selection arising through sex‐specific demographies—in particular, sex‐biased dispersal—may curb the evolution of male traits that harm females, including in the context of coevolutionary cycles of adaptation and counteradaptation of the two sexes. Rankin's analysis has stimulated further theory (Pizzari and Gardner 2012; Faria et al. 2015; Pizzari et al. 2015) and empirical research (Carazo et al. 2014), but this topic has been controversial (Chippindale et al. 2015; Hollis et al. 2015; Martin and Long 2015).

However, evolutionary conflicts of interest need not only occur between individuals, but may also occur within individuals (Haig 2000a; Burt and Trivers 2006). Indeed, kin selection has been implicated in fomenting intragenomic conflict, particularly in the context of sex‐specific demographies (Haig 2000b; Úbeda and Gardner 2010, 2011, 2012, 2015; Van Cleve et al. 2010; Brandvain et al. 2011; Gardner 2014; Úbeda et al. 2014; Farrell et al. 2015). For instance, if sex‐biased dispersal results in an individual being more related to their social partners through one parent than the other, their paternal‐ and maternal‐origin genes may disagree as to how selfishly the individual should behave (Haig 2000a; Úbeda and Gardner 2010). Such intragenomic conflict is expected to lead to parent of origin specific patterns of gene expression, that is, “genomic imprinting,” at evolutionary equilibrium (Haig and Westoby 1989). In particular, divergent selection on maternal‐ and paternal‐origin genes with respect to the level at which they are expressed is expected to lead to one gene being silenced, and the other to express at a level corresponding to its optimum. But whether this logic holds in the context of sexual conflict—and coevolutionary cycles—is unclear.

Here, we investigate the scope for sex‐biased dispersal to generate intragenomic conflict and drive the evolution of genomic architecture in relation to sexual‐conflict phenotypes. Specifically: we develop and analyze a kin‐selection model (Hamilton 1964; Taylor and Frank 1996; Gardner and Welch 2011; Gardner 2014) to determine the evolutionary interests of different classes of genetic actor involved in sexual conflict, that is, genes of maternal‐, paternal‐, and unknown‐origin; and we use the “loudest voice prevails” principle (Haig 1996; Úbeda and Haig 2004) to derive predictions as to patterns of gene expression and the consequences of mutations and epimutations of loci underpinning sexual‐conflict phenotypes.

## Model and Results

### MATHEMATICAL MODEL

Following Rankin (2011) and Faria et al. (2015), we consider an infinite diploid population divided into patches containing *n*_m_ males and *n*_f_ females, with multiple mating such that every female mates with every male in her patch, and vice versa. Males invest in a costly harming trait that increases personal reproductive success relative to other males in the patch, but reduces the overall fecundity of the females in the group. Females invest in a costly resistance trait, which reduces both the cost of being harmed and the benefit to the harming male. Specifically, a male's fecundity is $f_{m}=1+by\left(1-sx^{\prime }\right)-uy$, where *b* is the marginal fecundity gain of harming in the absence of resistance, *y* is his investment into harming, *s* is the impact of resistance, *x*′ is the average investment into resistance by females in his patch, and *u* is the marginal fecundity cost of investment into harming. Each male's relative reproductive success is proportional to his fecundity and inversely proportional to the average fecundity of the males in his patch. Likewise, a female's fecundity is $f_{f}=1-cy^{\prime }\left(1-hx\right)-vx$, where *c* is the marginal fecundity cost of being harmed in the absence of resistance, *y*′ is the average investment into harming by males in her patch, *h* is the effectiveness of resistance, *x* is her investment into resistance, and *v* is the marginal fecundity cost of investment into resistance. Without loss of generality, we set h=1, such that *x* is the proportion of the cost of being harmed recovered by a resisting female. Following mating, each female produces a large number of offspring, with an even sex ratio, in proportion to her fecundity. Adults then die, and males disperse with probability *m*_m_ and females disperse with probability *m*_f_ to a random patch, or else remain in their focal patch. Following dispersal, *n*_m_ males and *n*_f_ females survive at random within each patch to adulthood—all others perishing—returning the population to the beginning of the lifecycle.

### INTRAGENOMIC CONFLICT

#### Methodology

To assess intragenomic conflict, we employ the neighbor‐modulated fitness methodology of Taylor and Frank (1996). This determines when genes are associated with higher fitness, and hence favored by natural selection, directly via their impact on their bearer's phenotype and/or indirectly via the impact of identical by descent copies on their bearer's social partner's phenotypes. A key assumption of this approach is that there is vanishingly little genetic variation, such that selective differences in fitness are very small. The resulting conditions may be interpreted as describing when a gene's activity increases its inclusive fitness, such that we may formally describe each gene's optimum (Gardner and Welch 2011; Gardner 2014). Here, we take “gene” to mean a physical piece of DNA, as distinct from the “locus” at which such genes reside and the “allele” that describes that gene's type (Rousset 2004; Gardner and Welch 2011).

#### Male harm

We investigate the interests of different classes of genes in relation to the male‐harm phenotype by hypothetically granting control of this phenotype to a genic actor A, residing in the focal male's genome, and determining when an increase in harm increases the gene's inclusive fitness (Hamilton 1964; Gardner and Welch 2011; Gardner 2014). This occurs when:

(1) b1−sx¯−u1+by¯1−sx¯−uy¯−b1−sx¯−u1+by¯1−sx¯−uy¯r mm |A−c1−x¯1−cy¯1−x¯−vx¯r mm |A+r fm |A1−a>0,

where $\bar{x}$ is the population average level of resistance, $\bar{y}$ is the population average level of harm, r mm |A is the relatedness between the actor and the male's male patch mates, r fm |A is the relatedness between the actor and the male's female patch mates, and $a={(\left(1-m_{f}\right)}^{2}+{\left(1-m_{m}\right)}^{2})/2$ is the “scale of competition” in a viscous population with sex‐specific dispersal (Frank 1998; Gardner 2010; Faria et al. 2015; see Supporting Information for details). Increased investment into harm provides a direct‐fitness benefit for the actor by increasing the male's relative reproductive success (first term on left‐hand side of condition 1), but incurs an inclusive‐fitness loss by reducing the relative reproductive success of related mate competitors (second term) and reducing the overall fecundity of local females and of their mates (third term).

Condition (1) may be used to determine the convergence stable (Christiansen 1991; Taylor 1996; Davies et al. 2016) level of harm which, if it takes an intermediate value, is given by:

(2) yA∗=c(mf2−mf+mm2−mmr fm |A+r mm |A1−x+2u1−r mm |A1−vx−b1−r mm |A1−sx1−vxc21−r mm |A+mf2−mf+mm2−mmr fm |A+r mm |A1−xu−b1−sx

(see Supporting Information for details).

Inspection of condition (1) and equation (2) reveals that different genic actors may have different inclusive‐fitness interests in relation to harming, if they are differentially related to the male's patch mates (r mm |A and r fm |A). We consider three classes of genic actors in the male's genome: autosomal genes that lack parent‐of‐origin information (hereafter “ignorant genes,” A = I), autosomal genes that know themselves to have originated from the individual's mother (hereafter “maternal‐origin genes,” A = M), and autosomal genes that know themselves to have originated from the individual's father (hereafter “paternal‐origin genes,” A = P). If these genes exert some control over the phenotype and differ in their inclusive fitness interests, there is intragenomic conflict over that phenotype.

For ignorant genes (A = I), relatedness is given by:

(3) r mm |I=mm2−mm−mf2nf−1+nf1+2mm−2mf1−nf2−mm+nm1−mm2nf1−mm2+nm1−mf2+4−mf−mmmf+mmnfnm

and

(4) r fm |I=1−mf1−mmnf+nmnf1−mm2+nm1−mf2+4−mf−mmmf+mmnfnm

(see Supporting Information for details). For maternal‐origin genes (A = M), relatedness is given by:

(5) r mm |M=1nm+1−mm2nm−12−mf−mmnf−mm−mfnf−1+nm2+mf+mm3−mm−mf2nmnf1−mm2+nm1−mf2+4−mf−mmmf+mmnfnm;

and

(6) r fm |M=1−mf1−mm2−mf−mmnf−mm−mfnf−1+nm2+mf1−mm+mm3−mm2nf1−mm2+nm1−mf2+4−mf−mmmf+mmnfnm

(see Supporting Information for details). And for paternal‐origin genes (A = P), relatedness is given by:

(7) r mm |P=1nm+1−mm2nm−12nf+nm−mf2nf−1−mm3nm−2−nf−mmnm−1+mfnf3−mm−2−nm1−mm2nf1−mm2+nm1−mf2+4−mf−mmmf+mmnfnm

and

(8) r fm |P=1−mf1−mm2nf+nm−mf2nf−1−mm3nm−2−nf−mmnm−1+mfnf3−mm−nm1−mm−22(nf1−mm2+nm1−mf2+4−mf−mmmf+mmnfnm

(see Supporting Information for details). Note that relatedness for ignorant (I) genes is the average of relatedness for maternal‐origin (M) and paternal‐origin (P) genes (i.e., r mm |I= ½ r mm |M+ ½ r mm |P and r fm |I= ½ r fm |M+ ½ r fm |P), and is equivalent to relatedness for the focal male himself (Faria et al. 2015).

**Figure 1:**
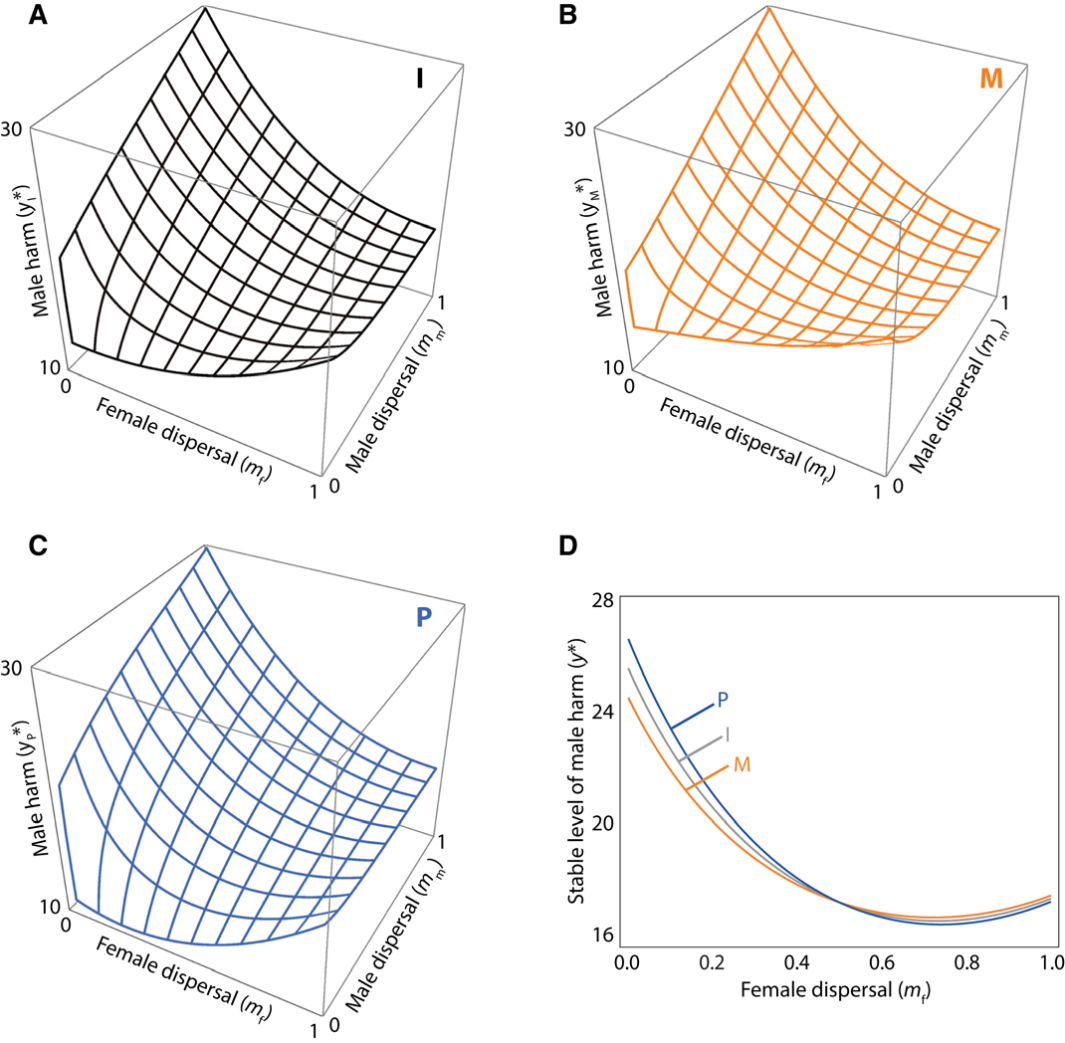
Intragenomic conflict over male harm in the absence of female resistance. Optimal level of male harm for: (A) a gene that is ignorant (I) of its parent‐of‐origin, (B) a gene that knows itself to be of maternal‐origin (M), and (C) a gene which knows itself to be of paternal‐origin (P). (D) Analytical predictions for ignorant control (gray), maternal‐origin control (orange), and paternal‐origin control (blue) for *m*_m_ = 0.50. In all panels, the other parameter values are *c* = 0.02, *b* = 0.05, *u* = 0.03, *n*_f_ = 3, and *n*_m_ = 3. [Color figure can be viewed at wileyonlinelibrary.com]

Substituting relatedness into equation (2) obtains the optimal level of harm for ignorant (Fig. 1A), maternal‐origin (Fig. 1B), and paternal‐origin genes (Fig. 1C). For illustration, assuming an even‐breeding sex ratio ($n_{f}=n_{m}$): if there is no sex bias in dispersal $\left(m_{f}=m_{m}\right)$, then all genic actors have the same relatedness to patch mates (r mm |M= r mm |I=r mm |P and r fm |M= r fm |I=r fm |P), that is, no intragenomic conflict ($y_{M}^{*}=y_{I}^{*}=y_{P}^{*}$; Fig. 1D); if dispersal is male‐biased ($m_{f}<m_{m}$) then relatedness is higher for maternal‐origin genes than for paternal‐origin genes (r mm |M>r mm |I>r mm |P and r fm |M>r fm |I>r fm |P), that is, potential for intragenomic conflict with maternal‐origin genes favoring less harm ($y_{M}^{*}<y_{I}^{*}<y_{P}^{*}$; Fig. 1D); and if dispersal is female‐biased ($m_{f}>m_{m}$), then relatedness is higher for paternal‐origin genes than for maternal‐origin genes (r mm |M<r mm |I<r mm |P and r fm |M<r fm |I<r fm |P), that is, potential for intragenomic conflict with paternal‐origin genes favoring less harm ($y_{M}^{*}>y_{I}^{*}>y_{P}^{*}$; Fig. 1D). In particular, if the male's harming is approximately optimal according to his own interests (or, equivalently, the interests of his ignorant genes; *y* ≈ y*_I_): then if dispersal is male‐biased, his maternal‐origin genes favor a reduction in harming (because y*_M_ < *y*) and his paternal‐origin genes favor an increase in harming (because y*_P_ > *y*); and if dispersal is female‐biased, his maternal‐origin genes favor an increase in harming (because y*_M_ > *y*) and his paternal‐origin genes favor a decrease in harming (because y*_P_ < *y*). To assess the correctness and robustness of these predictions, and for the purpose of concrete illustration, we develop an individual‐based simulation model tracking the evolution of harm under the control of maternal‐, paternal‐, or unknown‐origin genes, respectively (see Supporting Information for details). In each case, we find that the outcome is broadly consistent with the corresponding gene's optimum (Fig. S1), with the small discrepancies likely arising from mutation pressure and random drift—non‐Darwinian factors that are not considered in the analytical treatment.

#### Female resistance

Following the same procedure, we now consider the interests of a genic actor A = {M, P, I} carried by a focal female. We find that an increase in the female's resistance against harm increases the actor's inclusive fitness when:

(9) cy¯−v1−cy¯1−x¯−vx¯1−ar ff |A+1−ar mf |A>0,

where r ff |A is the relatedness between the actor and the female's female patch mates and r mf |A is the relatedness between the actor and the female's male patch mates (see Supporting Information for details; Faria et al. 2015). Owing to symmetrical, diploid inheritance, r fm |A = r mf |A. Because $c\bar{y}<1$, that is, fecundity cannot be negative, condition (9) is equivalent to $c\bar{y}>v$. Accordingly, irrespective of any differences in relatedness, the female's maternal‐, paternal‐, and unknown‐origin genes all favor increased resistance when the cost $c\bar{y}$ associated with harm exceeds the cost *v* of resistance, and these genes all favor reduced resistance when the reverse obtains, that is, there is no intragenomic conflict over resistance. Specifically, relatedness scales the strength of selection but does not affect its direction. This is because there is no trade‐off between a female's direct and indirect fitness: resistance increases her direct fitness and her male relatives’ fitness if it increases her fecundity, and it decreases her direct fitness and her male relatives’ fitness if it decreases her fecundity, so the necessary and sufficient criterion for an increase in female resistance to be favored is that it increases the female's fecundity, irrespective of degrees of relatedness (cf. Faria et al. 2015).

#### Coevolutionary cycles

Allowing harm and resistance to coevolve results in evolutionary cycling: increased harm favors increased resistance, which favors reduced harm and hence a concomitant reduction in resistance, etc. In contrast to the suggestion of Rankin (2011), repeated by Faria et al. (2015), we find that the selection dynamics involve unstable cycles that spiral inwards to asymptote at a stable equilibrium (see Supporting Information for details). However, these cycles may be long‐lasting and additional evolutionary forces–such as *de novo* mutation–may render them stable (see below). Interestingly, although sex‐biased dispersal leads to maternal‐ and paternal‐origin genes being favored to pull the male‐harm phenotype in different directions when at the equilibrium point corresponding to the ignorant genes’ interests (as above), this need not be true for all locations in trait space. In particular, it is often the case that both maternal‐ and paternal‐origin genes are favored to pull the male‐harm phenotype in the same direction while this phenotype is cycling around the equilibrium point (see Supporting Information and Fig. S2 for details).

### GENOMIC IMPRINTING

#### Loudest voice prevails

According to the kinship theory of genomic imprinting (Haig and Westoby 1989), and specifically the “loudest voice prevails” principle (Haig and Westoby 1989; Haig 1996; Úbeda and Haig 2004; Farrell et al. 2015), intragenomic conflicts such as that described above are predicted to lead to parent‐of‐origin specific gene expression and, in particular, the self‐imposed silencing of one of the genes at each conflicted locus. Considering a locus for which an increased gene expression leads to an increase in the contested phenotype (i.e., a “promoter” locus): then the gene with the larger phenotypic optimum may effect this by increasing its own expression and the gene with the lower phenotypic optimum may effect this by decreasing its own expression; each gene adjusting its expression in this way results in no net change in total gene expression at this locus, so further increases and decreases in gene expression are favored; and the evolutionary conflict ends with the gene with the lower phenotypic optimum silencing itself, such that the gene with the larger phenotypic optimum wins the intralocus conflict and sets its level of expression—and consequently, the level of the phenotype—according to its own optimum. Considering a locus for which an increased gene expression leads to a decrease in the contested phenotype (i.e., an “inhibitor” locus), then the logic is exactly reversed, and the gene with the larger phenotypic optimum is predicted to be silenced, whereas the gene with the lower phenotypic optimum is predicted to win the intralocus conflict and set its expression—and hence the phenotype—according to its optimum.

#### Male harm

**Figure 2:**
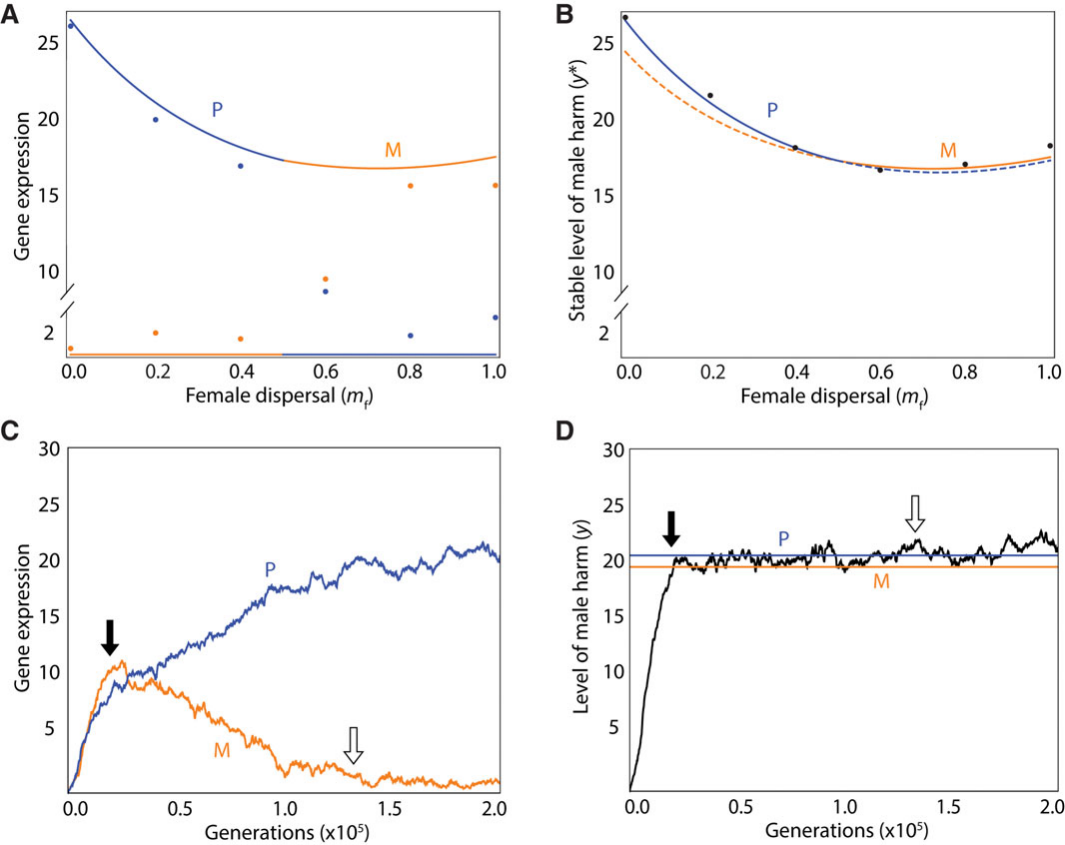
Genomic imprinting of a male‐harm promoter. (A) Analytical predictions (lines) and simulation results (dots, each representing a single replicate) for level of gene expression expected for the maternal‐origin gene (orange) and paternal‐origin gene (blue) at a locus whose gene product promotes male harm. (B) Resulting level of male harm, showing the interests of the gene (maternal‐ or paternal‐ origin) that wins the conflict (solid line) and the interests of the gene that loses the conflict (dashed line). (C) Evolution of gene expression of a male‐harm promoter for a maternal‐origin gene (orange) and paternal‐origin gene (blue). (D) Evolution of male harm, with the lines representing the optimal level for the maternal‐origin gene (orange) and paternal‐origin gene (blue). In all panels, the parameters are as follows: *c* = 0.02, *b* = 0.05, *u* = 0.03, *m*_m_ = 0.50, *n*_f_ = *n*_m_ = 3, with a mutation rate of 0.01 and 1000 patches. For panels (C) and (D), we indicate the generations in which intragenomic conflict initiates (i.e., male harm crosses into the zone between the two genetic optima; black arrow) and terminates (i.e., the maternal‐origin gene is effectively silenced, its expression decreasing to a value equal to its average over the final 2 × 10^5^ generations; white arrow), and we assume *m*_f_ = 0.20. [Color figure can be viewed at wileyonlinelibrary.com]

**Figure 3:**
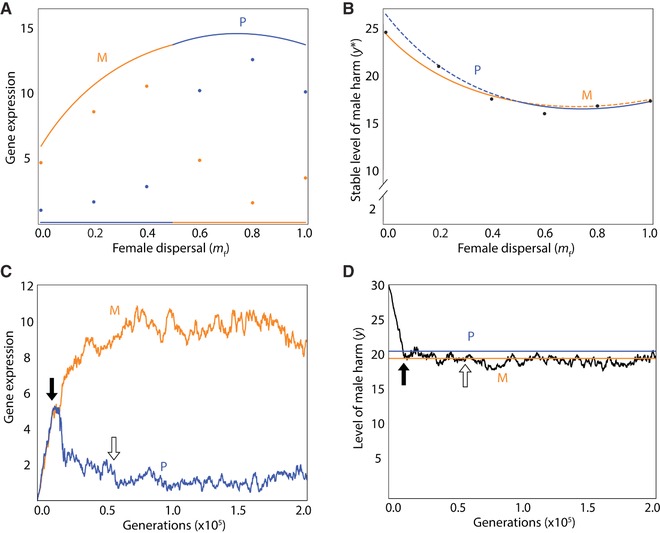
Genomic imprinting of a male‐harm inhibitor. (A) Analytical predictions (lines) and simulation results (dots, each representing a single replicate) for level of gene expression expected for the maternal‐origin gene (orange) and paternal‐origin gene (blue) at a locus whose gene product inhibits male harm. (B) Resulting level of male harm, showing the interests of the gene (maternal‐ or paternal‐origin) that wins the conflict (solid line) and the interests of the gene that loses the conflict (dashed line). (C) Evolution of genetic expression of a male‐harm inhibitor for a maternal‐origin gene (orange) and paternal‐origin gene (blue). (D) Evolution of male harm, with the lines representing the optimal level for the maternal‐origin gene (orange) and paternal‐origin gene (blue). In all panels, the parameters are as follows: *c* = 0.02, *b* = 0.05, *u* = 0.03, *m*_m_ = 0.50, *n*_f_ = *n*_m_ = 3, with a mutation rate of 0.01 and 1000 patches. For panels (C) and (D), we indicate the generations in which intragenomic conflict initiates (i.e., male harm crosses into the zone between the two genetic optima; black arrow) and terminates (i.e., the paternal‐origin gene is effectively silenced, its expression decreasing to a value equal to its average over the final 2 × 10^5^ generations; white arrow), and we assume *m*_f_ = 0.20. [Color figure can be viewed at wileyonlinelibrary.com]

We use the loudest voice prevails principle to derive predictions as to patterns of gene expression for conflicted loci underpinning the harm phenotype. Considering a harm‐promoter locus: then, if there is male‐biased dispersal ($m_{f}<m_{m}$), relatedness is higher for the maternal‐origin gene than for the paternal‐origin gene (r mm |M>r mm |P and r fm |M>r fm |P), leading to the maternal‐origin gene favoring the lower level of harm ($y_{M}^{*}<y_{P}^{*}$) and, accordingly, the maternal‐origin gene is predicted to be silenced and the paternal‐origin gene expressed at its optimal level (Fig. 2A), such that the level of harm is that which maximizes the paternal‐origin gene's inclusive fitness (Fig. 2B); and if there is female‐biased dispersal ($m_{f}>m_{m}$), the opposite pattern holds. Alternatively, considering a harm‐inhibitor locus: then, if there is male‐biased dispersal ($m_{f}<m_{m}$), relatedness is higher for the maternal‐origin gene than for the paternal‐origin gene (r mm |M>r mm |P and r fm |M>r fm |P), leading to the maternal‐origin gene favoring the lower level of harm ($y_{M}^{*}<y_{P}^{*}$) and, accordingly, the paternal‐origin gene is predicted to be silenced and the maternal‐origin gene expressed at its optimal level (Fig. 3A), such that the level of harm is that which maximizes the maternal‐origin gene's inclusive fitness (Fig. 3B); and, if there is female‐biased dispersal ($m_{f}>m_{m}$), the opposite pattern holds.

To assess the correctness and robustness of these predictions, and to provide a concrete illustration of the loudest voice prevails principle, we develop an individual‐based simulation model (see Supporting Information for details) tracking the evolution of parent‐of‐origin specific gene expression and the resulting male‐harm phenotype, until equilibrium is attained. Here, we consider harm evolving in the absence of resistance. Considering a male‐harm promoter (Fig. 2), and a population in which there is initially zero expression of either gene at this locus and hence no harming, we find that: imprinted gene expression does evolve, and in the expected direction—that is, the paternal‐origin gene expressing at a higher level under male‐biased dispersal, and the maternal‐origin gene expressing at a higher level under female‐biased dispersal—but with the less expressed gene being incompletely silenced, as a consequence of mutation‐selection balance (Fig. 2A); the resulting level of male harm is broadly consistent with the optimum for the expressed gene—that is, the paternal‐origin gene under male‐biased dispersal and the maternal‐origin gene under female‐biased dispersal (Fig. 2B).

Specifically, we find that both genes initially increase their expression (Fig. 2C), resulting in an increase in harming (Fig. 2D); until the phenotype surpasses the optimum for the gene favoring less harm (Fig. 2D, black arrow), after which time this gene's expression decreases, whereas the other gene's expression increases (Fig. 2C), until the former gene's expression reaches mutation‐selection balance close to zero (Fig. 2C, white arrow)—that is, it is effectively silenced—after which time the other gene attains its optimal level of expression (Fig. 2C) and optimal level of harm (Fig. 2D). Considering a male‐harm inhibitor, and a population in which there is initially zero expression of either gene at this locus and hence a maximal level of harm, recovers equivalent results (Fig. 3).

#### Female resistance

We showed above that there is no intragenomic conflict between a female's genes with respect to her resistance phenotype. Accordingly, the loudest voice prevails principle predicts no parent‐of‐origin specific gene expression for loci underpinning this phenotype. We use individual‐based simulations to explore the evolution of female resistance in the context of fixed levels of male harm, confirming that both maternal‐ and paternal‐origin genes favor the same level of female resistance—all or nothing, depending on the level of male harm—and finding no striking differences in each gene's level of expression (Fig. S3).

#### Coevolutionary cycles

Insofar as coevolution of harm and resistance results in the attainment of a stable equilibrium, in the region of the optimum for ignorant genes, sex‐biased dispersal is expected to lead to an intragenomic conflict of interests such that genes originating from one parent are favored to reduce harm and genes originating from the other parent are favored to increase harm. Accordingly, the loudest voice prevails principle predicts exactly the same patterns of parent‐of‐origin specific gene expression predictions as outlined above. However, the transient but nevertheless long‐lasting selection dynamics—and, potentially, the long‐term evolutionary dynamics—may involve cycling around the equilibrium and outwith the zone of intragenomic conflict. Consequently, it is unclear whether genomic imprinting is expected in this coevolutionary context.

**Figure 4:**
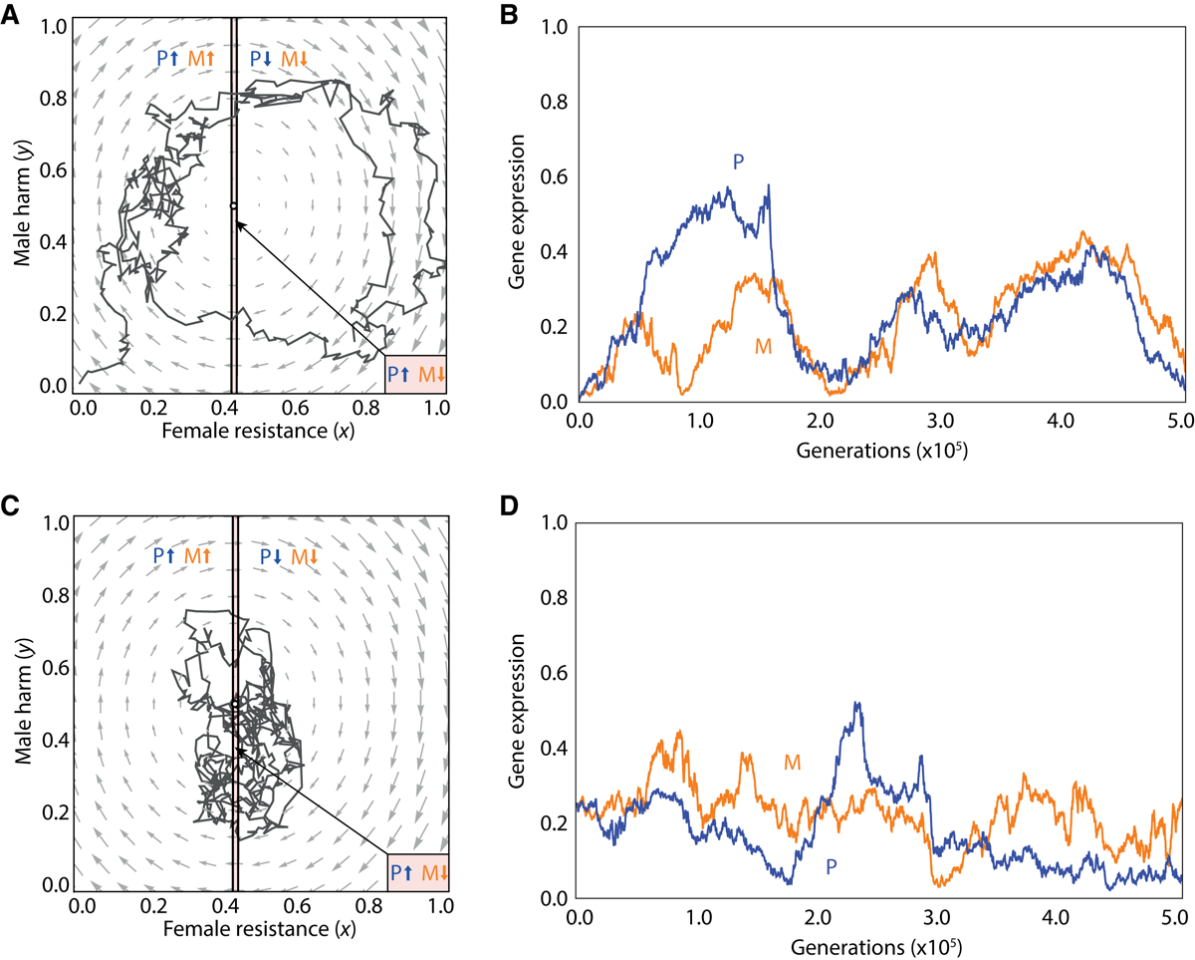
Cyclical coevolutionary dynamics of male harm *y* and female resistance *x* for a promoter of male harm. (A) Analytical predictions of the dynamics suggest that there is a stable point (black dot) where genomic imprinting may be present and to which the population arrives via an inward spiral from an initialization point at zero male harm, but individual‐based simulations additionally incorporating mutation and random drift (black lines) instead exhibit stable cycling. Arrows indicate direction (increase or decrease) of selection acting upon maternal‐origin (orange) and paternal‐origin (blue) genes, with the arrows pointing in opposite directions within the zone of conflict and pointing in the same direction outwith the zone of conflict. (B) Individual‐based simulation results for the level of expression of a promoter of male harm over multiple generations for maternal‐origin (orange) and paternal‐origin (blue) genes, when male harm is initialized at zero. (C) As in (A) but with the population initialized at its equilibrium level. (D) Individual‐based simulation results for the level of expression of a promoter of male harm over multiple generations for maternal‐origin (orange) and paternal‐origin (blue) genes, when the population initialized at its equilibrium level. We used the following values for the different parameters: *n*_f_ = *n*_m_ = 3, *c* = 0.02, *b* = 0.05, *u* = 0.03, *v* = 0.01, *s* = 0.75, *m*_f_ = 0, *m*_m_ = 0.5, with a mutation rate of 0.01 and 1000 patches. [Color figure can be viewed at wileyonlinelibrary.com]

**Figure 5:**
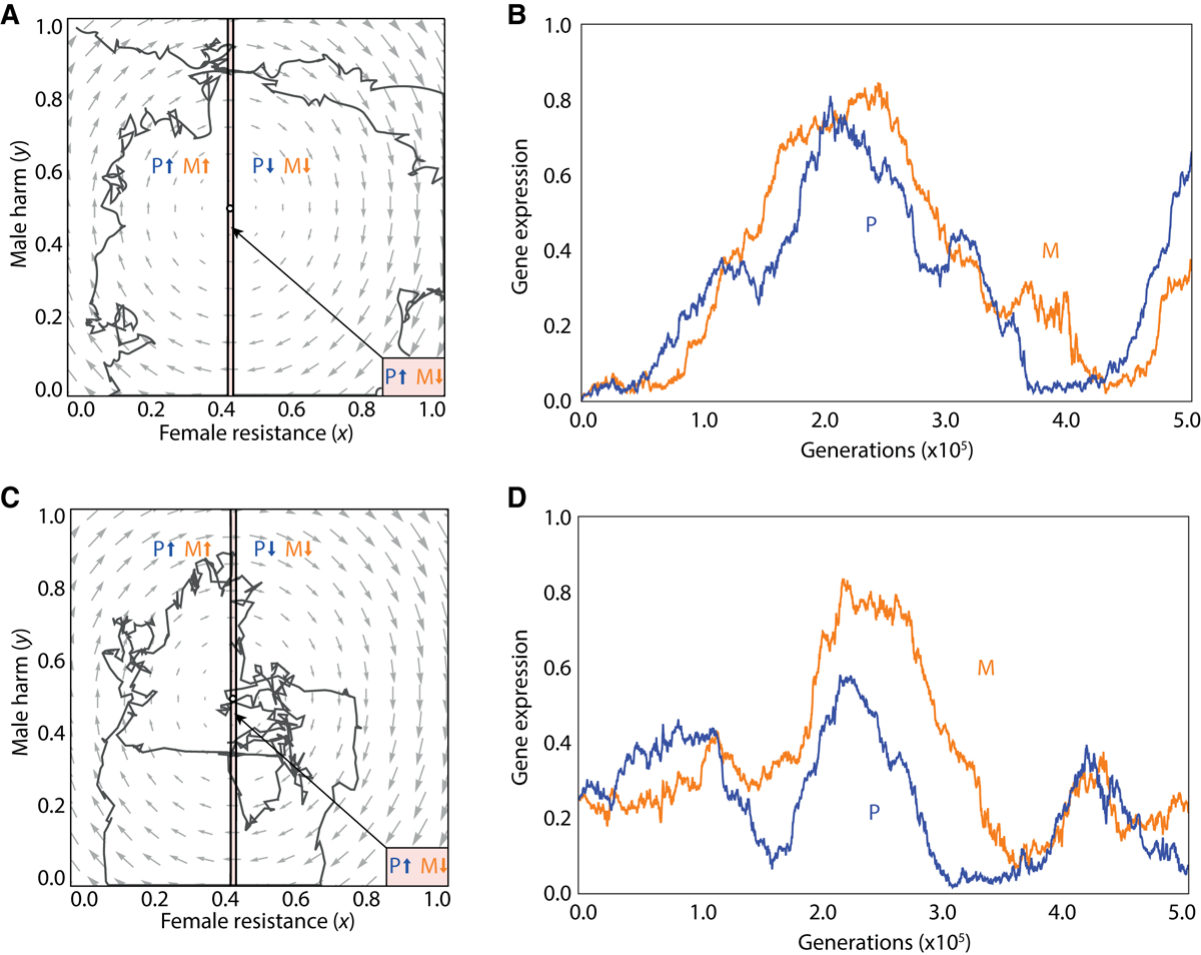
Cyclical coevolutionary dynamics of male harm *y* and female resistance *x* for an inhibitor of male harm. (A) Analytical predictions of the dynamics suggest that there is a stable point (black dot) where genomic imprinting may be present and to which the population arrives via an inward spiral from an initialization point at one of male harm, but individual‐based simulations additionally incorporating mutation and random drift (black lines) instead exhibit stable cycling. Arrows indicate direction (increase or decrease) of selection acting upon maternal‐origin (orange) and paternal‐origin (blue) genes, with the arrows pointing in opposite directions within the zone of conflict and pointing in the same direction outwith the zone of conflict. (B) Individual‐based simulation results for the level of expression of an inhibitor of male harm over multiple generations for maternal‐origin (orange) and paternal‐origin (blue) genes, when male harm is initialized at one. (C) As figure (A), but with the population initialized at its equilibrium level. (D) Individual‐based simulation results for the level of expression of an inhibitor of male harm over multiple generations for maternal‐origin (orange) and paternal‐origin (blue) genes, when the population initialized at its equilibrium level. We used the following values for the different parameters: *n*_f_ = *n*_m_ = 3, *c* = 0.02, *b* = 0.05, *u* = 0.03, *v* = 0.01, *s* = 0.75, *m*_f_ = 0, *m*_m_ = 0.5, with a mutation rate of 0.01 and 1000 patches. [Color figure can be viewed at wileyonlinelibrary.com]

We investigate this scenario using individual‐based simulations for the purpose of concrete illustration. Considering a male‐harm promoter, we find that coevolutionary cycles may arise irrespective of whether the population is initialized away from the equilibrium point (in particular, at zero harm and zero resistance; Fig. 4A and B) or at the equilibrium point (in particular, with harm and resistance set according to the interests of the ignorant genes; Fig. 4C and D), such that the cycles appear to be maintained indefinitely. Under both initialization treatments, the resulting cycles involve the population spending the majority of generations outwith the zone of conflict, such that both maternal‐ and paternal‐origin genes are typically favored to pull the phenotype in the same—albeit fluctuating—direction (Fig. 4A and C). Accordingly, although random genetic drift may lead to parent‐of‐origin specific gene expression, there is no clear direction to imprint (Fig. 4B and D). Consideration of a male‐harm inhibitor recovers equivalent results (Fig. 5). As above, we find no evidence of imprinting in relation to female resistance (Fig. S4).

### ASSOCIATED PATHOLOGIES

**Figure 6:**
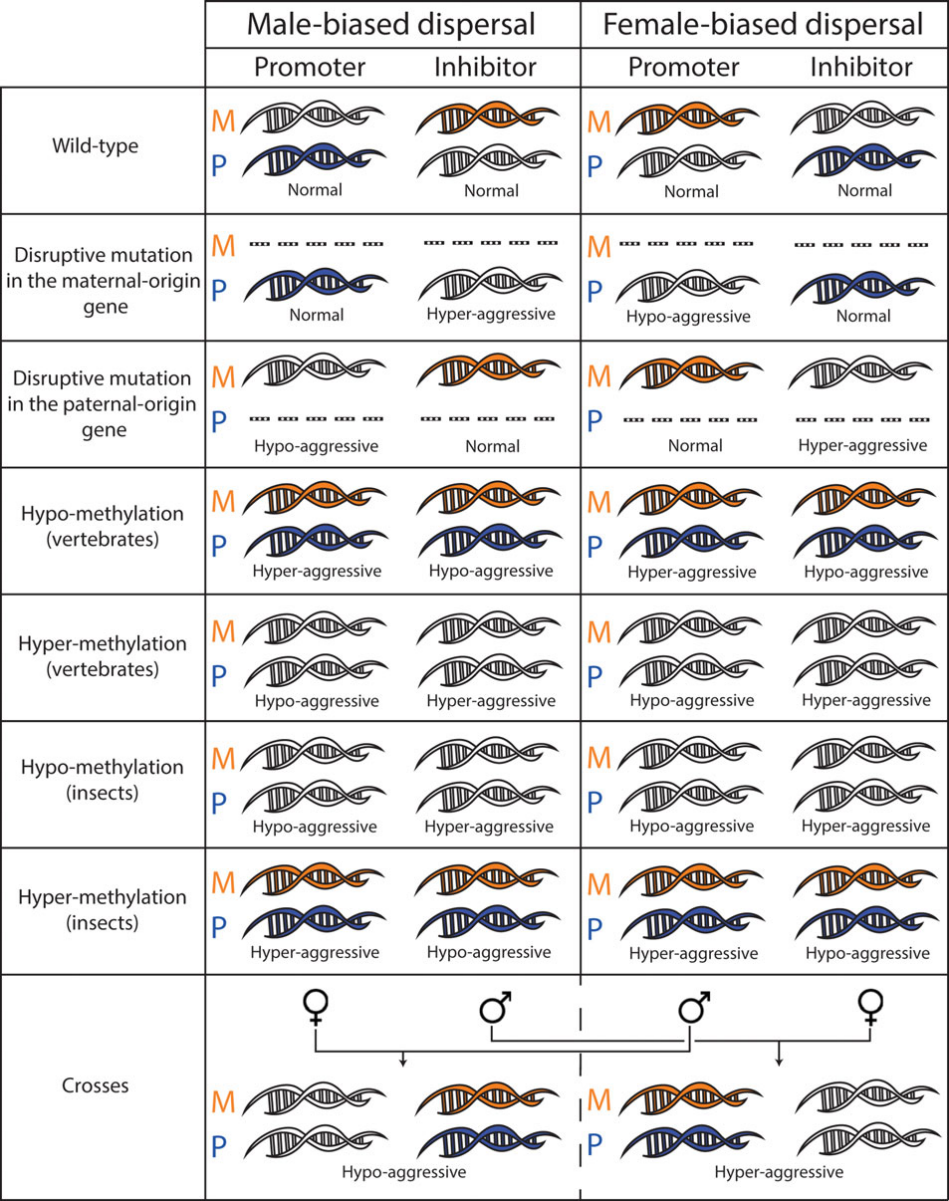
Expression of imprinted genes and effects of possible mutations (disruptive mutations, hypo‐methylation, or hyper‐methylation) under male‐ versus female‐biased dispersal, plus effects of crosses between females from populations with male‐biased dispersal and males from populations with female‐biased dispersal, and vice versa. Orange/blue genes are expressed, whereas colorless genes are silent. “Vertebrates” denotes all instances in which methylation leads to gene silencing and “insects” denotes all instances in which methylation leads to gene expression. [Color figure can be viewed at wileyonlinelibrary.com]

Genomic imprinting renders individuals functionally haploid and hence vulnerable to deleterious mutations (Holliday 1990). Indeed, in the context of human disease, mutations at imprinted loci are often associated with extreme pathologies (Hirasawa and Feil 2010), as the “tug of war” between paternal‐ and maternal‐origin genes—with each gene pulling the phenotype strongly in opposite directions as a consequence of even a slight discrepancy between their phenotypic optima—leads to a delicate equilibrium that, if the control exerted by one party is unexpectedly disrupted, may result in a phenotype that lies far beyond either gene's optimum. Such disruptions may include the following: mutations that result in a modification of the sequence coded by the DNA, and epimutations that modulate the presence/absence of methylation in critical zones of the genes. In either case, the phenotypic effect of such disruptions are expected to primarily manifest in males, as only this sex expresses the harming phenotype. Here, we outline predictions for the effects of such various disruptions to the normal expression of imprinted loci (Fig. 6).

A knock‐out/deletion mutation that results in a gene failing to produce any functional gene product has no phenotypic effect if that gene is silenced anyway. For instance, in the context of male‐biased dispersal: harm‐promoter loci are predicted to be maternally‐silenced and paternally‐expressed, such that a knockout/deletion of a maternal‐origin gene gives rise to a normal phenotype, but a knockout/deletion of a paternal‐origin gene results in a reduction in the harm‐promoting gene product and hence a hypo‐aggressive phenotype; and harm‐inhibitor loci are predicted to be paternally‐silenced and maternally‐expressed, such that a knockout/deletion of a maternal‐origin gene results in a reduction in the harm‐inhibiting gene product and hence a hyper‐aggressive phenotype but a knockout/deletion of a paternal‐origin gene results in a normal phenotype. Opposite predictions obtain in the context of female‐biased dispersal (Fig. 6).

Epimutations arise when changes occur in the normal pattern of DNA methylation, which is considered to be the main (though perhaps not exclusive; Bartolomei 2009) mechanism underpinning genomic imprinting. In vertebrates, DNA methylation is typically associated with gene silencing (Bird 2002), whereas in insects, it is typically associated with gene activation (Glastad et al. 2014). Accordingly, a disruption in methylation—whether it is accidental loss of methylation of a typically methylated gene (“hypo‐methylation”) or accidental methylation of a typically unmethylated gene (“hyper‐methylation”)—is expected to have different consequences for different taxa. In the context of taxa in which methylation tends to silence genes: hypo‐methylation of a harm‐promoter locus results in an increase in the harm‐promoting gene product and hence a hyper‐aggressive phenotype, whereas hyper‐methylation of a harm‐promoter locus results in a decrease in the harm‐promoting gene product and hence a hypo‐aggressive phenotype; and hypo‐methylation of a harm‐inhibitor locus results in an increase in the harm‐inhibiting gene product and hence a hypo‐aggressive phenotype, whereas hyper‐methylation of a harm‐inhibitor locus results in a decrease in the harm‐inhibiting gene product and hence a hyper‐aggressive phenotype. Conversely, we expect the opposite pattern in taxa in which methylation tends to activate genes (Fig. 6).

Another useful and practical way to study genomic imprinting is through reciprocal crosses between distinct populations or species (Haig and Westoby 1991; Oldroyd et al. 2013; Galbraith et al. 2016). For example, if a female of a species with female‐biased dispersal (i.e., expected to have maternally‐expressed harm‐promoter loci and paternally‐expressed harm‐inhibitor loci) is mated with a male of a species with male‐biased dispersal (i.e., expected to have paternally‐expressed harm‐promoter loci and maternally‐expressed harm‐inhibitor loci), then their male progeny are expected to have both maternal‐ and paternal‐origin genes expressed at harm‐promoter loci and to have both maternal‐ and paternal‐origin genes silenced at harm‐inhibitor loci, resulting in a hyper‐aggressive phenotype. In contrast, crosses between males from the first species and females from the second species are expected to yield hypo‐aggressive sons (Fig. 6).

## Discussion

We have investigated the potential for sex‐biased dispersal to generate intragenomic conflict and, consequently, drive the evolution of genomic imprinting in the context of sexual conflict. We have found that, insofar as sex‐specific demographies—such as sexual‐biased dispersal—lead to asymmetries in relatedness between members of mating groups with respect to their maternal‐ versus paternal‐origin genes, an intragenomic conflict of interest may arise that results in the evolution of genomic imprinting and increased vulnerability of individuals to mutational and epimutational challenge.

We expect intragenomic conflict to occur when individuals are more related to their social partners via one parent than via the other, such that their genes originating from one parent are more inclined toward kin‐selected selflessness with regards to these social partners than are the genes originating from the other parent (Haig 2000a; Úbeda and Gardner 2010). In the present study, selflessness manifests as a reduction in the extent to which a male harms his mating partners. Such intragenomic conflict between genes of maternal‐ versus genes of paternal‐origin is expected to drive parent‐of‐origin specific gene expression at loci underpinning male harm, with one gene typically silencing itself, while the other expresses at its optimal level (Haig 1996; Úbeda and Haig 2004). We predict that loci that promote male harm will be maternally‐silenced and paternally‐expressed when dispersal is male‐biased, on account of individuals being more related to their social partners via their mothers and hence their maternal‐origin genes being more inclined to selflessness; and we predict that loci that inhibit male harm will be paternally‐silenced and maternally‐ expressed, for the same reason. The same logic leads to exactly the opposite predictions when dispersal is female‐biased. In line with previous studies’ suggestions that genetic relatedness does not modulate the level of female resistance to male harm (Rankin 2011; Faria et al. 2015), we find no scope for intragenomic conflict or genomic imprinting with respect to this phenotype.

Following Rankin (2011) and Faria et al. (2015), we have considered a relatively generic male‐harm phenotype, which leads to a reduction in female fecundity and an increase in male relative reproductive success that we expect has application to a wide range of natural and experimental systems. Examples include aggressive behavior (Knott and Kohlenberg 2011; Feldblum et al. 2014), damaging genetalia (Crudgington and Siva‐Jothy 2000; Stutt and Siva‐Jothy 2001), toxic seminal proteins (Chapman et al. 1995; Chapman 2006), graspers (Arnqvist and Rowe 2002), and forced copulation (Brennan et al. 2010). We have also considered a relatively generic ecology and demography, which we expect has application to a wide range of species. In particular, although we have focused on sex‐biased dispersal, other forms of sex‐specific demography are expected to yield equivalent results (Úbeda and Gardner 2010, 2011, 2012). Moreover, following Rankin (2011), we have assumed that there are no deleterious consequences of consanguinous matings. Inbreeding depression may have a quantitative impact on the optima of conflicting genic agents, if elimination of some inbred individuals leads to an increase in average outbredness, with concomitant impact upon coefficients of relatedness, but it is not expected to alter our key, qualitative results. Importantly, our key predictions depend only on the existence and direction of intragenomic conflict, and not the magnitude of the discrepancy between optima: accordingly, we expect these patterns to be relatively robust to variation in model assumptions (Farrell et al. 2015).

However, not all taxa are equally likely to evolve genomic imprinting. Notably, *Drosophila melanogaster* lacks key methylation enzymes (Zemach et al. 2010; Raddatz et al. 2013) and consequently has very little methylation (Takayama et al. 2014) and limited scope for genomic imprinting. It has often been suggested that genomic imprinting is absent outwith mammals and flowering plants, largely on account of lack of evidence from model organisms such as *D. melanogaster* (Chapman 2006; Spencer and Clark 2014; Yan et al. 2014). Nevertheless, there is growing direct and indirect evidence of extensive methylation, and even genomic imprinting, in other insects, such as hymenoptera (Wang et al. 2006; Kronforst et al. 2008; Kucharski et al. 2008; Herb et al. 2012; Amarasinghe et al. 2014; Oldroyd et al. 2013; Yan et al. 2014, 2015; Cook et al. 2015; Galbraith et al. 2016; Remnant et al. 2016). Such taxa provide excellent opportunities for developing new theoretical and empirical avenues of genomic imprinting research (Queller 2003; Rautiala and Gardner 2016). More generally, while the current formulation of the kinship theory of genomic imprinting suggests that, whenever imprinting can evolve, it will evolve, provided there is a nonzero degree of conflict between maternal‐ versus paternal‐origin genes, more likely the extent of the conflict and hence selection for imprinting will need to exceed some nonzero threshold in order for it to evolve in the face of mutation pressure and other evolutionary forces. A better understanding of the factors acting in opposition to genomic imprinting is needed to explain why this phenomenon appears to be the exception rather than the rule (Wilkins et al. 2016).

It is unclear to what extent parent‐of‐origin specific gene expression modulates the phenotypic evolution of male‐harm traits at the individual level. Although a male‐harm phenotype underpinned by a single genetic locus may be expected to evolve to a quantitatively different level according to whether its gene product acts to promote or inhibit that phenotype—on account of this affecting which gene is expected to win the intragenomic conflict—more realistically, multigenic phenotypes may be expected to be underpinned by a roughly equal number of promoter and inhibitor loci and, the interests of maternal‐ and paternal‐origin genes more or less cancelling out, equilibrating at a level that is indistinguishable from the individual's inclusive‐fitness optimum (Grafen 2006; Gardner and Ross 2014; Farrell et al. 2015). Hence, the usual manifestation of this conflict of interest is likely limited to the molecular world rather than the traditional behavioral‐ecological realm of individuals and populations. The relative lack of exploration of the molecular underpinnings of sexually selected traits means that our theoretical predictions are made in the almost complete absence of empirical data on relevant patterns of genomic imprinting and, accordingly, this provides a valuable opportunity for a truly independent test of theory (Queller 2003; Wild and West 2009; Rautiala and Gardner 2016). Moreover, our predictions—namely, that male‐harm traits will have a tendency to be imprinted in structured populations—may facilitate the discovery of elusive genes underpinning such classic behavioral‐ecological phenotypes.

An important consequence of genomic imprinting is that the systematic silencing of the gene inherited from one parent renders the individual functionally haploid and, accordingly, vulnerable to the effects of disruptive mutations and epimutations at the imprinted locus. Moreover, the “tug of war” between maternal‐ and paternal‐origin genes that normally balances more or less at the individual's phenotypic optimum (but see Wilkins and Haig 2001; Wilkins 2010, 2011) may, in the event of one of these genetic parties being rendered nonfunctional, give rise to an extreme phenotype that is strongly maladaptive from either gene's perspective. Indeed, genomic imprinting has been implicated in a number of debilitating human diseases, including infant growth disorders (Hirasawa and Feil 2010), childhood cancers (Lim and Maher 2010), and possibly also neurological disorders such as autism and psychopathy (Crespi and Badcock 2008; Úbeda and Gardner 2010). Analogous to the hyper‐ and hypo‐aggressive sexual traits, we have predicted to be associated with particular classes of mutation and epimutation at imprinted loci underpinning male‐harm phenotypes, autism and psychopathy have been suggested to represent polar‐opposite phenotypes along a continuum of social‐brain disorders, with autism representing a hyperaltruistic brain (low cognitive empathy but high emotional empathy; Smith 2009; Úbeda and Gardner 2010) and psychopathy representing a hyperselfish brain (high cognitive empathy but low emotional empathy; McHoskey 2001; Úbeda and Gardner 2010).

These disorders may not be altogether independent of the male‐harm behaviors that have been the focus of our study, as selflessness in sexual conflict is simply one facet of a range of cooperative versus competitive behaviors that concern imprinted social brain theory. Indeed, the hitherto difficult‐to‐explain tendency for autism and psychopathy to be more prevalent in males than in females may point to their being manifestations of a breakdown of a normal male‐specific phenotype, like those involved in sexual conflict. Clear data are lacking regarding psychopathy, but some genes associated with autism have been reported to have male‐limited and parent‐of‐origin specific expression (Fradin et al. 2010; Corradi et al. 2014). More generally, we suggest that many behavioral polymorphisms—so‐called “animal personalities” (Wolf and Weissing 2012)—may be explicable with reference to gene‐level conflicts rather than having an adaptive rationale at the individual level.

We have also suggested that genomic imprinting effects may be observed in reciprocal crosses between distinct populations or species, with the production of either hyper‐ or hypo‐aggressive hybrids, depending on the dispersal patterns of each population or species. This could potentially affect the outcome of competitive interactions between hybridizing species. For instance, it may influence the outcome of competitive encounters, leading to narrowing or broadening of hybrid zones and deciding into which parental species’ territory the hybrid zone will expand, as well as with which parental species the hybrids will more often introgress. Hybridization has been reported to influence animal personalities, with hybrids having been described as exhibiting increased or decreased levels of aggression than either of their parental species in salmon (Einum and Fleming 1997), warblers (Pearson and Rohwer 2000), manakins (McDonald et al. 2001), and lizards (Robbins et al. 2014). We are not aware of such personality effects ever having been interpreted in the light of intragenomic conflict.

While our analysis provides support for the loudest voice prevails principle, which relates a mismatch between the inclusive fitness optima of maternal‐ versus paternal‐origin genes to patterns of parent‐of‐origin specific gene expression at evolutionary equilibrium—and, indeed, provides the first simulation exploration of the temporal unfolding of loudest voice prevails dynamics—it also highlights an important caveat that potentially limits the application of this principle to natural populations. In particular, we have found that in populations that are more‐or‐less permanently prevented from attaining evolutionary equilibrium—such as in the context of coevolutionary cycling—the phenotype may be sufficiently far from the optimum of either set of genes that these will be favored to drive the phenotype in the same direction, so that even when their optima do not exactly coincide they may nevertheless never come into actual intragenomic conflict. West and Gardner (2013) noted that the existence of intragenomic conflict provides strong support for the exquisite adaptedness of individuals, and here we reverse this principle by emphasizing that scenarios involving permanent maladaptation—for example, owing to fluctuating selection pressures induced by an antagonistic, coevolving party—may lead to the extinguishing of intragenomic conflicts. This may explain why, although there is extensive and growing evidence of imprinting across a wide range of organisms, not all loci that are otherwise expected to imprinted show parent‐of‐origin specific expression.

This is, as far as we are aware, the first study to have considered the kinship theory of genomic imprinting in relation to an explicitly sexually selected trait. Closely related topics that have been investigated from a parent‐of‐origin specific gene‐expression perspective include sex allocation (Queller 2003; Wild and West 2009; Haig 2014; Rautiala and Gardner 2016), sexual antagonism (Day and Bonduriansky 2004), paternal genome elimination (Gardner and Ross 2014), incest avoidance (Haig 1999), and alternation of asexual and sexual reproduction (Haig and Wilczek 2006). Although there has been significant interest in exploring the overlap and interplay of other forms of intragenomic conflict with mating success modulating behaviors, the emphasis of this research has been on understanding how selfish genes may drive the evolution of mating systems (Haig and Bergstrom 1995; Zeh and Zeh 1996; Price and Wedell 2008; Gardner and Ross 2011; Wedell 2013; Price et al. 2014; Taylor et al. 2014). In contrast, our analysis has reversed the direction of causation to consider how mating ecology has driven the evolution of intragenomic conflict. More generally, the present study has established further links between the theories of kin selection and sexual selection, two fields that have for a long time advanced almost completely independently of each other (Boomsma 2007; Rankin 2011; Wild et al. 2011; Pizzari and Gardner 2012; Pizzari et al. 2015). The present initial investigation of the overlap between the kinship theory of genomic imprinting and the evolution of sexual conflicts highlights an exciting opportunity for productive interplay between these two scientific realms.

Associate Editor: D. Roze

Handling Editor: M. Servedio

## Supporting information


**Figure S1**. Intragenomic conflict over male harm in the absence of female resistance.
**Figure S2**. Cyclical coevolutionary dynamics of male harm *y* and female resistance *x*.
**Figure S3**. Absence of genomic imprinting for female resistance traits.
**Figure S4**. Absence of clear genomic imprinting with respect to female resistance in coevolution with male harm.

Supporting Information
