# Supplementary material for: Sexual selection modulates genetic conflicts and patterns of genomic imprinting
Source: Evolution. 2017 Jan 16;71(3):526–40. doi: 10.1111/evo.13153 (PMC5347858; doi:10.1111/evo.13153)
Supplement: Supplementary file 1 — Figure S1. Intragenomic conflict over male harm in the absence of female resistance. Figure S2. Cyclical coevolutionary dynamics of male harm y and female resistance x. Figure S3. Absence of genomic imprinting for female resistance traits. Figure S4. Absence of clear genomic imprinting with respect to female resistance in coevolution with male harm. [file EVO-71-526-s001.pdf]

Supporting Information

(a) Natural selection

Natural selection will favour any gene that is associated with greater individual relative fitness, i. e. $\mathrm{d}W/\mathrm{d}g > 0$, where g is the genic value of a gene picked at random from the population and W is its carrier's expected, relative fitness (Taylor 1996). In the context of class structure, fitness must be averaged across individuals of different classes, e.g. $W = c_f W_f + c_m W_m$, where f and m denote female and male classes and c_f and c_m are the class reproductive values of females and males, respectively (Taylor 1996).

From Faria et al. (2015), female fitness in the context of the present model is given by:

$$W_{\mathrm{f}} = f_{\mathrm{f}}\left(\frac{1}{2}\left(\frac{1-m_{\mathrm{f}}}{(1-m_{\mathrm{f}})F_{\mathrm{f}}+m_{\mathrm{f}}\bar{F}_{\mathrm{f}}}+\frac{m_{\mathrm{f}}}{\bar{F}_{\mathrm{f}}}\right)+\frac{1}{2}\left(\frac{1-m_{\mathrm{m}}}{(1-m_{\mathrm{m}})F_{\mathrm{f}}+m_{\mathrm{m}}\bar{F}_{\mathrm{f}}}+\frac{m_{\mathrm{m}}}{\bar{F}_{\mathrm{f}}}\right)\right), \qquad \text{(A1)}$$

where $F_{\mathrm{f}} = f_{\mathrm{f}}|_{x=x'}$, $\bar{F}_f = f_{\mathrm{f}}|_{x=\bar{x},\ y'=\bar{y}}$, $F_m = f_{\mathrm{m}}|_{y=y'}$, and $\bar{F}_{\mathrm{m}} = f_{\mathrm{m}}|_{y=\bar{y},\ x'=\bar{x}}$. And male fitness is given by:

$$W_{\mathrm{m}} = \frac{f_{\mathrm{m}}}{F_{\mathrm{m}}}F_{\mathrm{f}}\left(\frac{1}{2}\left(\frac{1-m_{\mathrm{f}}}{(1-m_{\mathrm{f}})F_{\mathrm{f}}+m_{\mathrm{f}}\bar{F}_{\mathrm{f}}}+\frac{m_{\mathrm{f}}}{\bar{F}_{f}}\right)+\frac{1}{2}\left(\frac{1-m_{\mathrm{m}}}{(1-m_{\mathrm{m}})F_{\mathrm{f}}+m_{\mathrm{m}}\bar{F}_{\mathrm{f}}}+\frac{m_{\mathrm{m}}}{\bar{F}_{\mathrm{f}}}\right)\right) \qquad \text{(A2)}$$

Focusing upon a male-harm trait, we have:

$$\frac{\mathrm{d}W}{\mathrm{d}g} = c_{\mathrm{f}}\frac{\partial W_{\mathrm{f}}}{\partial y'}\frac{\mathrm{d}y'}{\mathrm{d}G'_{\mathrm{m}}}\frac{\mathrm{d}G'_{\mathrm{m}}}{\mathrm{d}g_{\mathrm{f|A}}} + c_{\mathrm{m}}\left(\frac{\partial W_{\mathrm{m}}}{\partial y}\frac{\mathrm{d}y}{\mathrm{d}G_{\mathrm{m}}}\frac{\mathrm{d}G_{\mathrm{m}}}{\mathrm{d}g_{\mathrm{m|A}}} + \frac{\partial W_{\mathrm{m}}}{\partial y'}\frac{\mathrm{d}y'}{\mathrm{d}G'_{\mathrm{m}}}\frac{\mathrm{d}G'_{\mathrm{m}}}{\mathrm{d}g_{\mathrm{m|A}}}\right) \qquad \text{(A3)}$$

where: G_m is the focal male's breeding value; G'_m is the averaging breeding value of the males of the focal patch; $\mathrm{d}y/\mathrm{d}G_m = \mathrm{d}y'/\mathrm{d}G'_m = \psi$ is the genotype-phenotype map; $\mathrm{d}G_m/\mathrm{d}g_{m|A} = p_{m|A}$ is the consanguinity of the genic actor A in the focal male to the male himself (Bulmer 1994); $\mathrm{d}G'_m/\mathrm{d}g_{m|A} = p_{mm|A}$ is the consanguinity of the genic actor A in the focal male with a randomly-chosen male on his patch (including the focal male himself); $\mathrm{d}G'_m/\mathrm{d}g_{f|A} = p_{fm|A}$ is the consanguinity of the genic actor A in the focal male to a randomly-chosen female on his patch; and $c_f = c_m = ½$ under diploidy (Taylor 1996). The relatedness between the genic actor A in the focal male and a randomly-chosen male on his patch (including the focal male himself) may then be described as $r_{mm|A} = p_{mm|A}/p_{m|A}$ and the relatedness between the actor A in the focal male and a randomly-chosen female on his patch by $r_{fm|A} = p_{fm|A}/p_{m|A}$ (Bulmer 1994).

For the female resistance trait, we have:

$$\frac{\mathrm{d}W}{\mathrm{d}g} = c_{\mathrm{f}}\left(\frac{\partial W_{\mathrm{f}}}{\partial x}\frac{\mathrm{d}x}{\mathrm{d}G_{\mathrm{f}}}\frac{\mathrm{d}G_{\mathrm{f}}}{\mathrm{d}g_{\mathrm{f|A}}} + \frac{\partial W_{f}}{\partial x'}\frac{\mathrm{d}x'}{\mathrm{d}G'_{\mathrm{f}}}\frac{\mathrm{d}G'_{\mathrm{f}}}{\mathrm{d}g_{\mathrm{f|A}}}\right) + c_{\mathrm{m}}\frac{\partial W_{\mathrm{m}}}{\partial x'}\frac{\mathrm{d}x'}{\mathrm{d}G'_{\mathrm{f}}}\frac{\mathrm{d}G'_{\mathrm{f}}}{\mathrm{d}g_{\mathrm{m|A}}} \qquad \text{(A4)}$$

where: G_f is a focal female's breeding value; and G'_f is the averaging breeding value of the females of the focal patch; $\mathrm{d}x/\mathrm{d}G_f = \mathrm{d}x'/\mathrm{d}G'_f = \xi$ is the genotype-phenotype map; $\mathrm{d}G_f/\mathrm{d}g_{f|A} = p_{f|A}$ is the consanguinity of the genic actor A in the focal female to the female herself (Bulmer 1994); $\mathrm{d}G'_f/\mathrm{d}g_{f|A} = p_{ff|A}$ is the consanguinity of the genic actor A in the focal female with a randomly-chosen female on her patch (including the focal female herself); and $\mathrm{d}G'_f/\mathrm{d}g_{m|A} = p_{fm|A}$ is the consanguinity of the genic actor A in the focal female to a random male on her

patch. We can then express the relatedness between the genic actor A in the focal female to a randomly-chosen female on her patch (including the focal female herself) by $r_{\mathrm{ff|A}} = p_{\mathrm{ff|A}}/p_{\mathrm{f|A}}$ (Bulmer 1994). The consanguinity between a genic actor A to its carrier is the same no matter the genic actor or the sex that we are considering and, therefore, $p_{\mathrm{f|A}} = p_{\mathrm{m|A}} = p$ and, accordingly, the relatedness between a genic actor and its carrier is always 1.

(b) Relatedness

The relatedness between a genic actor A in the focal male with a randomly-chosen male in his patch (including the focal male himself) is:

$$r_{\mathrm{mm|A}} = \frac{1}{n_{\mathrm{m}}} + \frac{n_{\mathrm{m}}-1}{n_{\mathrm{m}}}(1-m_{\mathrm{m}})^2 r_{\mathrm{A}}, \quad \text{(A5)}$$

where: with probability of $1/n_{\mathrm{m}}$ the randomly-chosen male is the focal male himself, in which case relatedness is 1; and with probability $(n_{\mathrm{m}}\text{-}1)/n_{\mathrm{m}}$ is a different male, in which case they are only related if they are both locals $(1\text{-}m_{\mathrm{m}})^2$ and, if so, their relatedness is defined by the relatedness through the genic actor A (r_{A}) in the focal male. For the relatedness between a genic actor A in the focal male with a random female in his patch:

$$r_{\mathrm{fm|A}} = (1-m_{\mathrm{f}})(1-m_{\mathrm{m}})r_{\mathrm{A}}, \quad \text{(A6)}$$

and they are only related if they are both locals $(1\text{-}m_{\mathrm{m}})(1\text{-}m_{\mathrm{f}})$ and, if so, their relatedness is defined by the relatedness through the genic actors A (r_{A}) in the focal male. For the relatedness between a genic actor A in the focal female with another random female in her patch (including the focal female herself):

$$r_{\mathrm{ff|A}} = \frac{1}{n_{\mathrm{f}}} + \frac{n_{\mathrm{f}}-1}{n_{\mathrm{f}}}(1-m_{\mathrm{f}})^2 r_{\mathrm{A}} \quad \text{(A7)}$$

where: with probability of $1/n_{\mathrm{f}}$ it is drawn the focal female herself, in which case the relatedness is 1; and with probability $(n_{\mathrm{f}}\text{-}1)/n_{\mathrm{f}}$ is a different male, in which case they are only related if they are both locals $(1\text{-}m_{\mathrm{f}})^2$ and, if so, their relatedness is defined by the relatedness through the genic actors A (r_{A}) in the focal female. For the relatedness between a genic actor A in the focal female with a random male in his patch:

$$r_{\mathrm{mf|A}} = (1-m_{\mathrm{m}})(1-m_{\mathrm{f}})r_{\mathrm{A}}, \quad \text{(A8)}$$

and they are only related if they are both locals $(1\text{-}m_{\mathrm{m}})(1\text{-}m_{\mathrm{f}})$ and, if so, their relatedness is defined by the relatedness through the genic actors A (r_{A}) in the focal female.

Relatedness through the genic actor A between two different juveniles born in the same patch is then given by $r_{\mathrm{A}} = p'_{\mathrm{A}}/p$ (Bulmer 1994), where p'_{A} is the consanguinity through the genic actor A between two individual born in the same patch and is defined by picking the genic actor A the focal male and a random gene from the other individual and calculating the probability that the two are identical by descent (Bulmer 1994). Focusing upon ignorant genes (A = I) and assuming that consanguinities are at their neutral-equilibrium values (which is appropriate if selection is weak (Gardner et al. 2011);), we write:

$$p'_{\mathrm{I}} = \frac{1}{4}\left(\frac{1}{n_{\mathrm{f}}}p + \frac{n_{\mathrm{f}}-1}{n_{\mathrm{f}}}(1-m_{\mathrm{f}})^2 p'_{\mathrm{U}}\right) + \frac{1}{4}\left(\frac{1}{n_{\mathrm{m}}}p + \frac{n_{\mathrm{m}}-1}{n_{\mathrm{m}}}(1-m_{\mathrm{m}})^2 p'_{\mathrm{U}}\right) + \frac{1}{2}(1-m_{\mathrm{f}})(1-m_{\mathrm{m}})p'_{\mathrm{U}} \quad \text{(A9)}$$

That is: with probability of ¼ we may have drawn the maternal-origin genes from both individuals, in which case with probability of $1/n_f$ they share the same mother (and they have consanguinity of p) and with probability of $(n_f-1)/n_f$ they have different mothers (and they will only have consanguinity if both mothers are local, giving a consanguinity of $(1-m_f)^2 p'_I$); with probability of ¼ we may have drawn the paternal-origin genes from both offspring, in which case with probability of $1/n_m$ they share the same father (and they have consanguinity of p) and with probability of $(n_m-1)/n_m$ they have different fathers (and they will only have consanguinity if both fathers are local, giving a consanguinity of $(1-m_m)^2 p'_I$); and with probability of ½ we have drawn the maternal-origin gene from one and the paternal-origin gene from the other and they will only have consanguinity if both these parents are locals (giving a consanguinity of $(1-m_f)(1-m_m)p'_I$). Rearranging equation (A5), we obtain:

$$p'_{\mathrm{I}} = \frac{n_{\mathrm{f}}+n_{\mathrm{m}}}{(1-m_{\mathrm{f}})2n_{\mathrm{m}}+(1-m_{\mathrm{m}})2n_{\mathrm{f}}+(4-m_{\mathrm{f}}-m_{\mathrm{m}})(m_{\mathrm{f}}+m_{\mathrm{m}})n_{\mathrm{f}}n_{\mathrm{m}}} p. \quad \text{(A10)}$$

Relatedness between two random individuals born in the same patch is then given by $r_I = p'_I/p$ (Bulmer 1994). Rearranging, we obtain:

$$r_{\mathrm{I}} = \frac{n_{\mathrm{f}}+n_{\mathrm{m}}}{(1-m_{\mathrm{f}})^2 n_{\mathrm{m}}+(1-m_{\mathrm{m}})^2 n_{\mathrm{f}}+(4-m_{\mathrm{f}}-m_{\mathrm{m}})(m_{\mathrm{f}}+m_{\mathrm{m}})n_{\mathrm{f}}n_{\mathrm{m}}} \quad \text{(A11)}$$

The consanguinity between two juveniles is given by:

$$p'_{\mathrm{I}} = \frac{1}{2}(p'_{\mathrm{M}} + p'_{\mathrm{P}}), \quad \text{(A12)}$$

and, by its turn:

$$p'_{\mathrm{M}} = \frac{1}{2}\left(\frac{1}{n_{\mathrm{f}}}p + \frac{n_{\mathrm{f}}-1}{n_{\mathrm{f}}}(1-m_{\mathrm{f}})^2 p'_{\mathrm{I}}\right) + \frac{1}{2}(1-m_{\mathrm{f}})(1-m_{\mathrm{m}})p'_{\mathrm{I}} \quad \text{(A13)}$$

$$p'_{\mathrm{P}} = \frac{1}{2}\left(\frac{1}{n_{\mathrm{m}}}p + \frac{n_{\mathrm{m}}-1}{n_{\mathrm{m}}}(1-m_{\mathrm{m}})^2 p'_{\mathrm{I}}\right) + \frac{1}{2}(1-m_{\mathrm{f}})(1-m_{\mathrm{m}})p'_{\mathrm{I}}. \quad \text{(A14)}$$

Relatedness between two random individuals in the same patch through their maternal-origin genes is then given by $r_M = p'_M/p$ (Bulmer 1994) and through their paternal-origin genes by $r_P = p'_P/p$ (Bulmer 1994). Rearranging, we obtain:

$$r_{\mathrm{M}} = \frac{(2-m_{\mathrm{f}}-m_{\mathrm{m}})(n_{\mathrm{f}}-m_{\mathrm{m}}-m_{\mathrm{f}}(n_{\mathrm{f}}-1))+(2+m_{\mathrm{f}}(1-m_{\mathrm{m}})+(3-m_{\mathrm{m}})m_{\mathrm{m}})n_{\mathrm{m}}}{2(1-m_{\mathrm{m}})^2 n_{\mathrm{f}}+2(1-m_{\mathrm{f}})^2 n_{\mathrm{m}}+2(4-m_{\mathrm{f}}-m_{\mathrm{m}})(m_{\mathrm{f}}+m_{\mathrm{m}})n_{\mathrm{f}}n_{\mathrm{m}}} \quad \text{(A15)}$$

$$r_{\mathrm{P}} = \frac{2(n_{\mathrm{f}}+n_{\mathrm{m}})-m_{\mathrm{f}}^2(n_{\mathrm{f}}-1)-m_{\mathrm{m}}(3n_{\mathrm{m}}-2-n_{\mathrm{f}}-m_{\mathrm{m}}(n_{\mathrm{m}}-1))+m_{\mathrm{f}}((3-m_{\mathrm{m}})n_{\mathrm{f}}+n_{\mathrm{m}}(m_{\mathrm{m}}-1)-2)}{2(1-m_{\mathrm{m}})^2 n_{\mathrm{f}}+2(1-m_{\mathrm{f}})^2 n_{\mathrm{m}}+2(4-m_{\mathrm{f}}-m_{\mathrm{m}})(m_{\mathrm{f}}+m_{\mathrm{m}})n_{\mathrm{f}}n_{\mathrm{m}}} \quad \text{(A16)}$$

Substituting (A15) and (A16) into expressions (A5) and (A6) obtains equations (5), (6), (7), and (8) of the main text.

(c) Stable levels of male harm

Substituting $\bar{y} = 0$ into the left-hand side of inequality (1) and seeing when the condition is satisfied determines when the male-harm optimum for genic actor A is greater than zero. In the event that the optimum is greater than zero, its value may be found by setting the left-hand side of inequality (1) equal to zero and solving for $\bar{y} = y_{\mathrm{A}}^*$. Accordingly, we find that genic actor A's male-harm optimum is given by:

$$y_{\mathrm{A}}^{*} = \begin{cases} \frac{c(m_{\mathrm{f}}(2-m_{\mathrm{f}})+m_{\mathrm{m}}(2-m_{\mathrm{m}})(r_{\mathrm{fm|A}}+r_{\mathrm{mm|A}})(1-x)+2(u(1-r_{\mathrm{mm|A}})(1-vx)-b(1-r_{\mathrm{mm|A}})(1-sx)(1-vx))}{c(2(1-r_{\mathrm{mm|A}})+(m_{\mathrm{f}}(2-m_{\mathrm{f}})+m_{\mathrm{m}}(2-m_{\mathrm{m}}))(r_{\mathrm{fm|A}}+r_{\mathrm{mm|A}}))(1-x)(u-b(1-sx))}, & b(r_{\mathrm{mm|A}})(1-sx) > a\frac{c(r_{\mathrm{fm|A}}+r_{\mathrm{mm|A}})(1-x)+2u(1-r_{\mathrm{mm|A}})(1-vx)}{1-vx} \\ 0, & b(r_{\mathrm{mm|A}})(1-sx) < a\frac{c(r_{\mathrm{fm|A}}+r_{\mathrm{mm|A}})(1-x)+2u(1-r_{\mathrm{mm|A}})(1-vx)}{1-vx} \end{cases} \quad \text{(A13)}$$

As the left-side of inequality (1) is a monotonically-decreasing function of $\bar{y}$, there is only one possible convergence-stable level of male harm (Davies et al. 2016; Christiansen 1991; Taylor 1996).

<u>(d) Stability analysis of the cycles</u>
The Jacobian matrix of our model is then given by:

$$\mathrm{J(a)} = \begin{pmatrix} \nabla W_x(a) \\ \nabla W_y(a) \end{pmatrix}, \quad \text{(A14)}$$

where $\nabla W_x(a)$ and $\nabla W_y(a)$ are the partial derivatives at $a = \{x, y\}$. The equilibrium point (x^*, y^*) is stable if $\mathrm{Re}(\lambda) < 0$, where λ is the leading eigenvalue of J. Numerical exploration suggests this equilibrium, when it exists, is always stable, and it is certainly the case for the specific parameter set (i.e. $n_{\mathrm{f}} = n_{\mathrm{m}} = 3$, $c = 0.02$, $b = 0.05$, $u = 0.03$, $v = 0.01$, $s = 0.75$, $m_{\mathrm{f}} = 0$ and $m_{\mathrm{m}} = 0.5$) considered in this study.

<u>(e) Individual-based simulation</u>
We run individual-based simulations where we consider an initial population of 1000 patches (*pat* = 1000) in which each patch contains three males and three females ($n_{\mathrm{m}} = n_{\mathrm{f}} = 3$). Each individual has a probability of being a parent to the individuals of the next generation and that probability is given by their fecundity. However, rather than giving rise to offspring, we jump straight to new adults. Each of the new adults has a probability of being from a specific patch, being this dependent on the dispersal rates of each sex as well on the fecundity present in each patch. Then, we assign to each one of the adults a mother and a father from the patch where they came from (which may be different from the patch where the individual is now, if she has dispersed). The gene value, transmitted from the parents to the adults of the next generation, may change due to mutations (either increasing or decreasing) which add up to the original value of the gene. The range of that change varies between -0.25 and 0.25 for the simulations where only male harm is present and between -0.01 and 0.01 for the simulations where female resistance is also present. In both cases, we are using a uniform distribution to modulate the mutational changes that occur in the traits considered. The only constraint to the values of the traits is that they cannot decrease below 0 (for all) or increase above 0.5 (for Female_Resistance_Promoter and Female_Resistance_Inhibitor). For all the simulations, this happens with a probability of 10^{-2}. These values will then affect the level of harm and/or the level of female resistance. The level of male harm is defined in different ways in different simulations: controlled by the sum of the two genes (Ignorant_Harm, Harm_Promoter, Promoter_Cycle, Promoter_CycleOptimal); controlled by the maternal gene (Maternal_Harm); or controlled by the paternal gene (Paternal_Harm). In all of these, an increase in the genes' values leads to an increase in male harm. On the contrary, in the Harm_Inhibitor we assumed an initial level of harm of 30 which can be reduced by the sum of the genes values. The same happens in Inhibitor_Cycle and Inhibitor_CycleOptimal but the initial level of harm is now 1. The level of female resistance is also defined in a similar way: controlled by the sum of the two genes (Promoter_Cycle, Promoter_CycleOptimal, Female_Resistance_Promoter, Inhibitor_Cycle, and Inhibitor_CycleOptimal) which leads to an increase of the level of the trait; or the sum of the two genes reduces the level of female resistance already present in the population, in this case an initial level of 1

(Female_Resistance_Inhibitor). The simulations were run for 10^5 generations for the Ignorant_Harm, Maternal_Harm, and Paternal_Harm, for 2 x 10^5 generations for Female_Resistance_Promoter and Female_Resistance_Inhibitor, and for 5 x 10^5 generations for all the others. The simulation's dots present in Figures 2-3, S1, and S3 are always the mean of the last 10000 generations and the genes' expression dynamics, represented in Figures 2C-D, 3C-D, and S4, report the first 2 x 10^5 generations.

Supplementary Figures

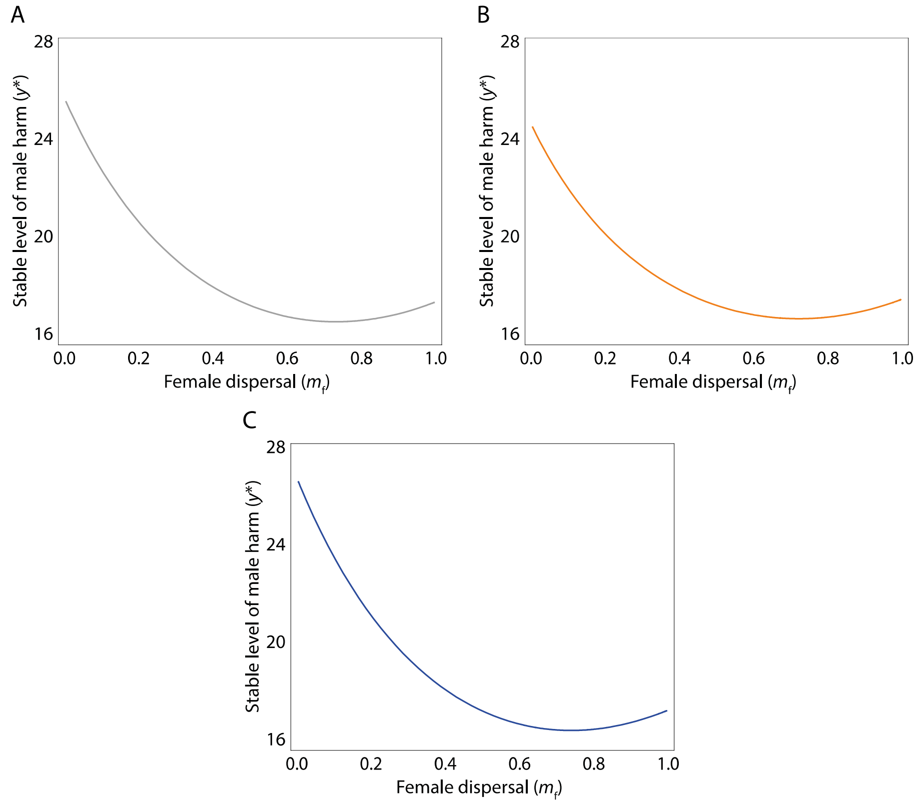

Figure S1 | Intragenomic conflict over male harm in the absence of female resistance. Analytical predictions (lines) and individual-based simulation results (dots, each representing a single replicate) for ignorance of parent-of-origin (gray – ***A***), maternal-origin control (orange – ***B***) and paternal-origin control (blue – ***C***), for m_m = 0.50. In all panels, the other parameter values are c = 0.02, b = 0.05, u = 0.03, n_f = n_m = 3, with a mutation rate of 0.01 and 1000 patches.

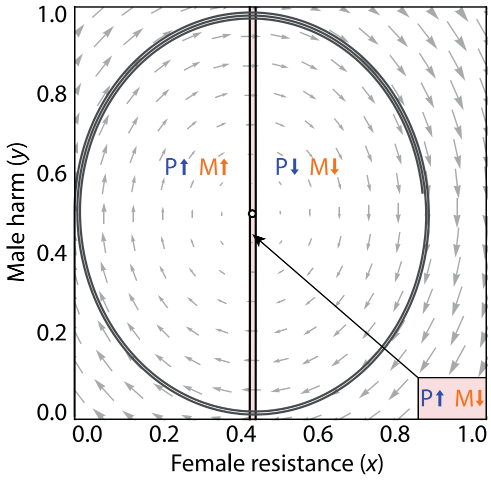

Figure S2 | Cyclical coevolutionary dynamics of male harm *y* and female resistance *x*. Analytical predictions of the dynamics suggest that there is a stable point (black dot) where genomic imprinting may be present and to which the population arrives via an inward spiral from an initialization point at zero male harm. Arrows indicate direction (increase or decrease) of selection acting upon maternal-origin (orange) and paternal-origin (blue) genes, with the arrows pointing in opposite directions within the zone of conflict and pointing in the same direction outwith the zone of conflict. We used the following values for the different parameters: $n_f = n_m = 3$, $c = 0.02$, $b = 0.05$, $u = 0.03$, $v = 0.01$, $s = 0.75$, $m_f = 0$, and $m_m = 0.5$.

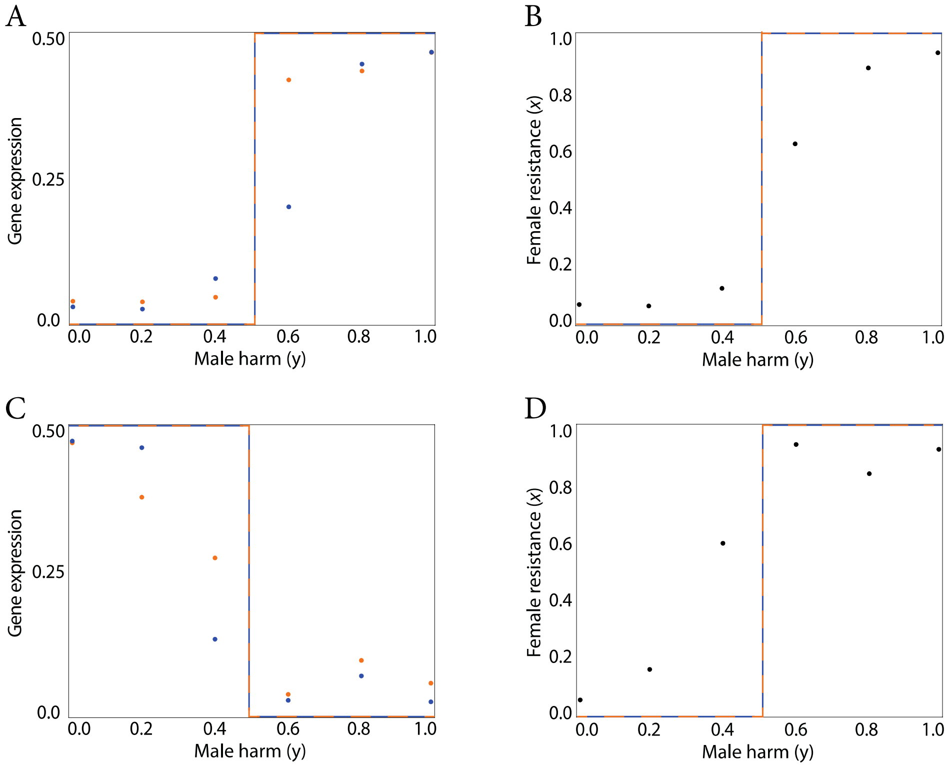

Figure S3 | Absence of genomic imprinting for female resistance traits. ***A***, Analytical predictions (lines) and simulation results (dots, each representing a single replicate) for level of gene expression expected for the maternal-origin gene (orange) and paternal-origin gene (blue) at a locus whose gene product promotes female resistance. ***B***, Resulting level of female resistance. ***C***, Analytical predictions (lines) and simulation results (dots, each representing a single replicate) for level of gene expression expected for the maternal-origin gene (orange) and paternal-origin gene (blue) at a locus whose gene product inhibits female resistance. ***D***, Resulting level of female resistance. In all panels, the parameters are: $c = 0.02$, $m_f = 0$, $m_m = 0.50$, $n_f = n_m = 3$, with a mutation rate of 0.01 and 1000 patches.

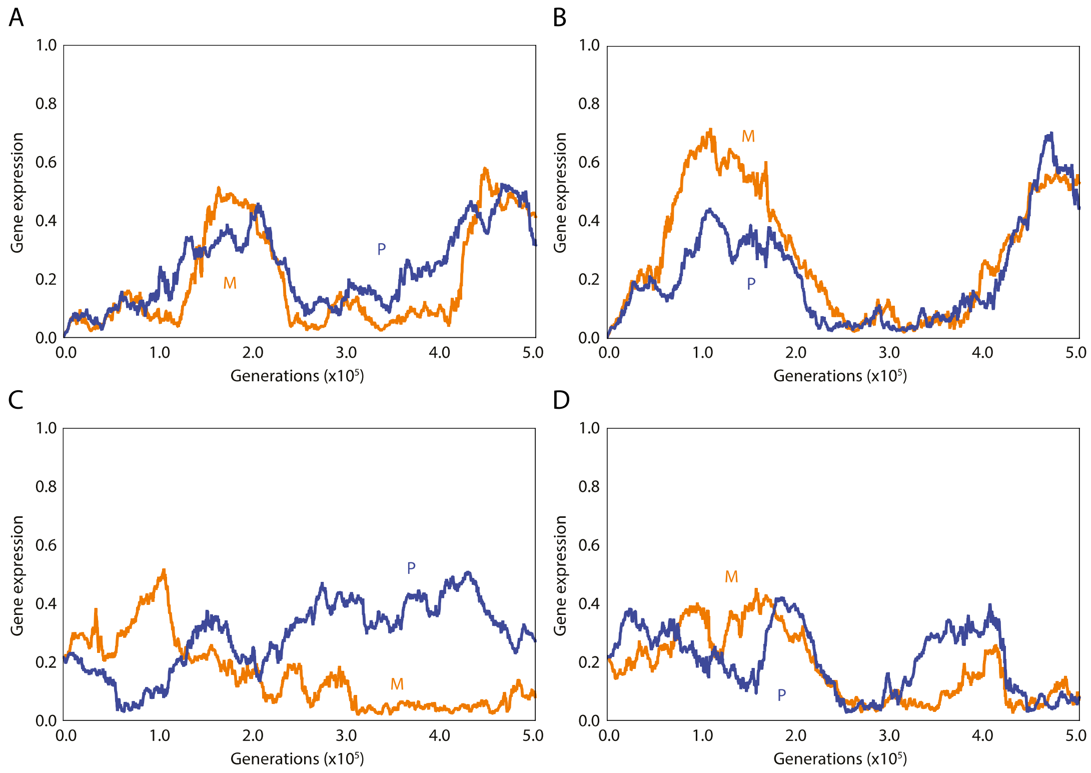

Figure S4 | Absence of clear genomic imprinting with respect to female resistance in co-evolution with male harm. ***A***, Individual-based simulation results for the level of expression of a promoter of female resistance over multiple generations for maternal-origin (orange) and paternal-origin (blue) genes, when a promoter of male harm and male harm itself is initialized at zero. ***B***, Individual-based simulation results for the level of expression of a promoter of female resistance over multiple generations for maternal-origin (orange) and paternal-origin (blue) genes, when an inhibitor of male harm is initialized at zero and male harm at one. ***C***, Individual-based simulation results for the level of expression of a promoter of female resistance over multiple generations for maternal-origin (orange) and paternal-origin (blue) genes, when the population initialized at its equilibrium level and the male harm gene is a promoter. ***D***, Individual-based simulation results for the level of expression of a promoter of female resistance over multiple generations for maternal-origin (orange) and paternal-origin (blue) genes, when the population initialized at its equilibrium level and the male harm gene is an inhibitor. We used the following values for the different parameters: $n_f = n_m = 3$, $c = 0.02$, $b = 0.05$, $u = 0.03$, $v = 0.01$, $s = 0.75$, $m_f = 0$, $m_m = 0.5$, with a mutation rate of 0.01 and 1000 patches.

References:

Bulmer, M. G. 1994. Theoretical Evolutionary Ecology. Sinauer Associates, Sunderland, MA.

Christiansen, F. B. 1991. On Conditions for Evolutionary Stability for a Continuously Varying Character. Am. Nat. 138:37–50.

Davies, N. G., L. Ross, and A. Gardner. 2016. The ecology of sex explains patterns of helping in arthropod societies. Ecol. Lett. 19(8):862-72.

Faria, G. S., S. A. M. Varela, and A. Gardner. 2015. Sex-biased dispersal, kin selection and the evolution of sexual conflict. J. Evol. Biol. 28:1901–1910.

Gardner, A., S. A. West, and G. Wild. 2011. The genetical theory of kin selection. J. Evol. Biol. 24:1020–1043.

Taylor, P. D. 1996. Inclusive fitness arguments in genetic models of behaviour. J. Math. Biol. 34:654–674.
